# Phosphorylation of FtsZ and FtsA by a DNA Damage-Responsive Ser/Thr Protein Kinase Affects Their Functional Interactions in *Deinococcus radiodurans*

**DOI:** 10.1128/mSphere.00325-18

**Published:** 2018-07-18

**Authors:** Ganesh K. Maurya, Kruti Modi, Manisha Banerjee, Reema Chaudhary, Yogendra S. Rajpurohit, Hari S. Misra

**Affiliations:** a Molecular Biology Division, Bhabha Atomic Research Centre, Mumbai, India; b Homi Bhabha National Institute (DAE—Deemed University), Mumbai, India; University of Iowa

**Keywords:** DNA damage response, *Deinococcus*, Ser/Thr protein kinase, bacterial cell cycle regulation, protein phosphorylation, radioresistance

## Abstract

Deinococcus radiodurans, a highly radioresistant bacterium, does not show LexA-dependent regulation of *recA* expression in response to DNA damage. On the other hand, phosphorylation of DNA repair proteins such as PprA and RecA by a DNA damage-responsive Ser/Thr protein kinase (STPK) (RqkA) could improve their DNA metabolic activities as well as their roles in the radioresistance of D. radiodurans. Here we report RqkA-mediated phosphorylation of cell division proteins FtsZ and FtsA *in vitro* and in surrogate Escherichia coli bacteria expressing RqkA. Mass spectrometric analysis mapped serine 235 and serine 335 in FtsZ and threonine 272, serine 370, and serine 386 in FtsA as potential phosphorylation sites. Although the levels of FtsZ did not change during postirradiation recovery (PIR), phosphorylation of both FtsZ and FtsA showed a kinetic change during PIR. However, in an *rqkA* mutant of D. radiodurans, though FtsZ underwent phosphorylation, no kinetic change in phosphorylation was observed. Further, RqkA adversely affected FtsA interaction with FtsZ, and phosphorylated FtsZ showed higher GTPase activity than unphosphorylated FtsZ. These results suggest that both FtsZ and FtsA are phosphoproteins in D. radiodurans. The increased phosphorylation of FtsZ in response to radiation damage in the wild-type strain but not in an *rqkA* mutant seems to be regulating the functional interaction of FtsZ with FtsA. For the first time, we demonstrate the role of a DNA damage-responsive STPK (RqkA) in the regulation of functional interaction of cell division proteins in this bacterium.

**IMPORTANCE** The LexA/RecA-type SOS response is the only characterized mechanism of DNA damage response in bacteria. It regulates cell cycle by attenuating the functions of cell division protein FtsZ and inducing the expression of DNA repair proteins. There are bacteria, including Deinococcus radiodurans, that do not show this classical SOS response. D. radiodurans is known for its extraordinary resistance to gamma radiation, and a DNA damage-responsive Ser/Thr protein kinase (RqkA) has been characterized for its role in radioresistance. RqkA phosphorylates a large number of proteins in solution. The phosphorylation of RecA and PprA by RqkA enhanced their activities. FtsZ phosphorylation is inducible by gamma radiation in wild-type D. radiodurans but not in an *rqkA* mutant. Phosphorylation affected the interaction of FtsZ and FtsA in this bacterium. This study, therefore, brought forth some findings that might lead to the discovery of a new mechanism regulating the bacterial cell cycle in response to DNA damage.

## Abstract (precis)

The LexA/RecA-type SOS response is the only characterized mechanism of DNA damage response in bacteria. It regulates cell cycle by attenuating the functions of cell division protein FtsZ and inducing the expression of DNA repair proteins. There are bacteria, including Deinococcus radiodurans, that do not show this classical SOS response. D. radiodurans is known for its extraordinary resistance to gamma radiation, and a DNA damage-responsive Ser/Thr protein kinase (RqkA) has been characterized for its role in radioresistance. RqkA phosphorylates a large number of proteins in solution. The phosphorylation of RecA and PprA by RqkA enhanced their activities. FtsZ phosphorylation is inducible by gamma radiation in wild-type D. radiodurans but not in an *rqkA* mutant. Phosphorylation affected the interaction of FtsZ and FtsA in this bacterium. This study, therefore, brought forth some findings that might lead to the discovery of a new mechanism regulating the bacterial cell cycle in response to DNA damage.

## INTRODUCTION

Bacterial cell division and genome segregation are interdependent processes regulated by the coordinated action of two macromolecular complexes, the divisome and the segrosome (1). Divisome components are nearly conserved across the bacteria (2) and have been found in all sequenced bacterial genomes. Cell division has been studied in both pathogenic and nonpathogenic bacteria, including Escherichia coli, Bacillus subtilis, Caulobacter crescentus, Mycobacterium tuberculosis, and Streptococcus pneumoniae (2, 3). FtsZ is known to be an integral part of the divisome complex and plays a pivotal role in bacterial cell division. The interacting partners of FtsZ and the underlying molecular mechanisms of divisome assembly are better understood in rod-shaped bacteria, e.g., E. coli and B. subtilis (4). Recently, the interdependence of cell division and genome segregation in the regulation of bacterial growth has been reviewed and a number of regulatory proteins working at the crossroads of these processes have been highlighted (5). Under normal growth conditions, the functions of FtsZ are influenced by a number of proteins such as FtsA, ZipA, ZapA, and EzrA and by proteins that inhibit minicell formation (minicell chromosomal deletion mutant [MinCDE] system) and by nucleoid occlusion systems such SlmA in E. coli and Noc in B. subtilis (6–9). In response to DNA damage, FtsZ polymerization and its GTPase activity are inhibited by SOS response proteins such as SulA in E. coli (10), YneA in Bacillus subtilis (11), DivS in Corynebacterium glutamicum (12), and Rv2719c in M. tuberculosis (13). Interestingly, there are some bacteria that do not confer a LexA-mediated classical SOS response. In such bacteria, the mechanisms underlying cell cycle regulation, particularly in response to DNA damage, are not clearly understood. In eukaryotes, however, the serine/threonine (S/T) phosphorylation of both regulatory and structural proteins and their involvement in cell cycle regulation have been demonstrated (14). Recently, S/T phosphorylation has also been shown in some bacterial cell division proteins, and the roles of such a process in the regulation of cell division and genome maintenance have been highlighted (15, 16).

Deinococcus radiodurans exhibits extraordinary resistance to DNA-damaging agents, including radiation and desiccation (17, 18). This bacterium displays efficient DNA repair mechanisms (19) and protects its other biomolecules, particularly proteins, from oxidative damage (20, 21). D. radiodurans harbors multipartite genome composed of 2 chromosomes and 2 plasmids. Cross talk between divisome and genome maintenance proteins has been demonstrated in this bacterium (22). In D. radiodurans, like other bacteria, cell division is arrested in response to DNA damage. The mechanism of this arrest, however, remains unknown. In other bacteria, SOS response proteins bring about cell division arrest in response to DNA damage (K. Modi, unpublished data). D. radiodurans, however, does not show a classical LexA/RecA-mediated SOS response (23, 24), and its genome does not encode the homologues of known SOS response cell division regulatory proteins. However, the genome of this bacterium encodes a large number of Ser/Thr protein kinases (STPKs) (25). One such Ser/Thr protein kinase (named "RqkA") from D. radiodurans has been characterized, and its role in radiation resistance has been demonstrated (26). The amino acid sequences of a large number of deinococcal proteins, including some of the cell division and genome maintenance proteins, showed the presence of putative phosphorylation sites (-S/T-Q-X-hydrophobic-hydrophobic- [where "S/T" represents the phosphoacceptor and X can be any amino acid except the positively charged residue]) (27) for eukaryote-like Ser/Thr protein kinases (eSTPKs). The phosphorylation of DNA repair proteins such as RecA and PprA (a pleiotropic protein involved in DNA repair in *Deinococcus*) by RqkA and the subsequent improvement in their role(s) in the radioresistance of this bacterium have been demonstrated (28, 29). Cell division proteins such as FtsZ and FtsA of D. radiodurans are 35% and 46% identical to homologues in E. coli, respectively. Recently, FtsZ of D. radiodurans was characterized as a slow GTPase which was stimulated by its cognate partner FtsA *in vitro* (30, 31). The levels of FtsZ do not change during postirradiation recovery (PIR), but the growth is arrested with a longer lag phase than was seen with an unirradiated control (30). Therefore, the molecular mechanism(s) regulating cell cycle arrest and FtsZ function in response to γ-radiation damage would be worth understanding in this radioresistant bacterium, which apparently lacks the typical canonical LexA/RecA-type SOS response mechanism.

Here, we report the phosphorylation of FtsZ and FtsA by a native DNA damage-responsive STPK (RqkA) of D. radiodurans and the role of phosphorylation in the regulation of FtsZ’s functional interaction with cognate FtsA in this bacterium. We demonstrated that in D. radiodurans, both FtsZ and FtsA underwent phosphorylation and the phosphorylation was radiation inducible and changed during PIR in the wild-type strain. RqkA could phosphorylate these proteins *in vitro*, and the phosphorylation of FtsZ could affect its GTPase activity as well as its interaction with FtsA both *in vitro* and *in vivo*. On the other hand, phosphorylation of FtsA did not result in any detected change in its interaction with FtsZ and P-FtsZ. Interestingly, in the *rqkA* mutant, the FtsZ phosphorylation did not result in any detected kinetic change during PIR and did not affect the interaction between these proteins. This indicated involvement of RqkA in regulation of cell division in response to DNA damage. Thus, for the first time, we report the involvement of a DNA damage-responsive Ser/Thr protein kinase, e.g., RqkA, in the phosphorylation of two important cell division proteins and its effect on their interactions in D. radiodurans.

## RESULTS

### RqkA phosphorylates FtsA and FtsZ of Deinococcus radiodurans* in vitro*.

**FIG 1:**
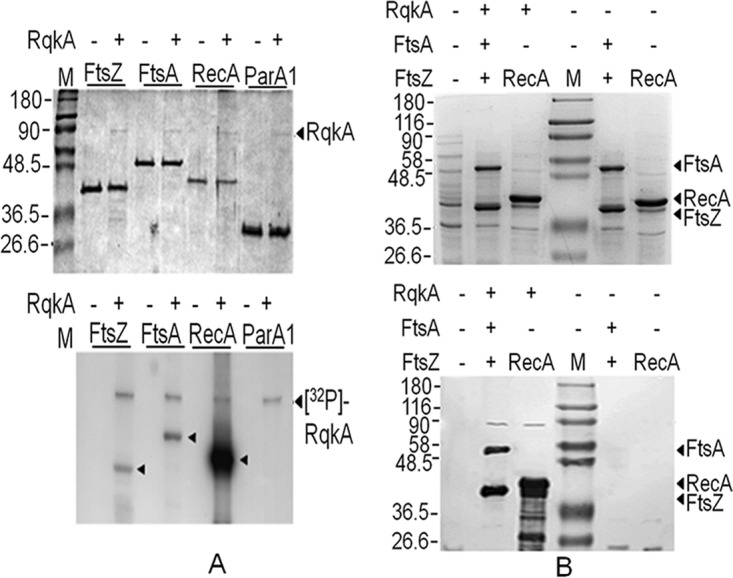
*In vitro* phosphorylation of cell division proteins of Deinococcus radiodurans by a DNA damage-responsive protein kinase (RqkA). Purified recombinant FtsZ, FtsA, RecA, and chromosome I ParA (Para1) (2 µM) were incubated with a 25×-lower molar concentration of purified RqkA in the presence of [γ-^32^P]ATP. Mixtures were separated on SDS-PAGE and dried, and signals were developed on autoradiogram (A). RecA was used as the positive control, and Para1 was used as the negative control. For phosphorylation studies inside the bacterial cells, the surrogate E. coli BL21 cells harboring RqkA expression plasmid (+) and corresponding vector (-) were cotransformed with plasmids expressing FtsA along with FtsZ. E. coli harboring empty vector (-) and recombinant plasmids expressing RqkA and RecA were used as controls. Total proteins from these cells were separated on SDS-PAGE and immunoblotted using phospho-Ser/Thr (immunoblot [anti-p-Ser/Thr]) epitope antibodies as described in Materials and Methods. Bands corresponding to FtsA (P-FtsA), RqkA (P-RqkA), and FtsZ variants (P-FtsZ) are marked in the immunoblots (lower panel) (B). Sizes of immunostained protein bands were estimated using molecular weight markers (M). Arrows indicate the identity and position of the respective phosphoprotein bands. Data given are representative of results from the reproducible experiments repeated 3 times.

The phosphorylation of FtsZ and FtsA by RqkA was monitored with RecA as a positive control (29) and with ParA1, which apparently does not contain a putative phosphorylation site(s) for STPKs, as a negative control. The purified RqkA phosphorylated the recombinant FtsA, FtsZ, and RecA but not Para1 (Fig. 1A). In order to ascertain the involvement of phosphorylation in the regulation of FtsZ functions, the phosphorylation status of FtsZ and FtsA when present together was monitored in surrogate E. coli cells expressing RqkA of D. radiodurans. Both FtsZ and FtsA from the cells expressing RqkA in *trans* were found to have been phosphorylated (Fig. 1B). The phosphorylation site(s) in FtsZ and FtsA phosphorylated by RqkA *in vitro* was analyzed using mass spectrometry. The results showed the phosphorylation of FtsZ at serine 235 and serine 335 sites (see Fig. S1 in the supplemental material), while FtsA was phosphorylated at threonine 272, serine 370, and serine 386 sites (Fig. S2). Although further studies on the functional significance of these phosphorylation sites are pending, the available results suggested that RqkA phosphorylates FtsZ and FtsA *in vitro* and in surrogate hosts expressing both these proteins with RqkA.

10.1128/mSphere.00325-18.1 FIG S1  Mass spectrometric analysis of phospho-FtsZ. Purified FtsZ was incubated with lowest molar ratio of purified RqkA (25:1 FtsZ/RqkA), and phosphorylated protein was subjected to mass spectrometric analysis in the Taplin Biological Mass Spectrometry Facility at Harvard Medical School, Boston, MA. Data were compared with the data from FtsZ processed identically without RqkA. The 2 phosphopeptides corresponding to serine 235 and serine 335 were detected as phosphorylation sites with ion scores of 45 and 49, respectively. Detailed data may be accessed using the following information: the user identifier (ID) is “msguest,” and the password was “spectrum.” At this site, we found peptides identified from our samples and their matches with corresponding proteins in the database. Data generated from both of the samples were compared. FtsZ control did not produce any phosphorylated peptide, while the peptides with phosphorylation sites (indicated with a number symbol [#]) were obtained from P-FtsZ. Based on the analysis and ion scores, the indicated peptides with phosphorylation sites (#) were confidently assigned. Download FIG S1, PDF file, 0.3 MB. Copyright © 2018 Maurya et al. 2018 Maurya et al. This content is distributed under the terms of the Creative Commons Attribution 4.0 International license.

10.1128/mSphere.00325-18.2 FIG S2  Peptide mass fingerprint of FtsZ interacting phosphoprotein (FIPP) excised from SDS-PAGE gel of immunoprecipitates of FtsZ antibodies. Cell extracts of D. radiodurans cells exposed to gamma radiation were immunoprecipitated using FtsZ antibodies, and precipitates were analyzed on SDS-PAGE and stained with Coomassie brilliant blue. The protein band was excised, and its peptide mass fingerprint (PMF) was obtained using mass spectrometry. Panel A shows identified posttranslational modification (PTM), and panel B shows mass spectra of identified protein FIPP as deinococcal FtsA. Download FIG S2, DOC file, 1.3 MB. Copyright © 2018 Maurya et al. 2018 Maurya et al. This content is distributed under the terms of the Creative Commons Attribution 4.0 International license.

### FtsZ and FtsA are phosphorylated *in vivo*, and their interaction shows a kinetic change during PIR.

**FIG 2:**
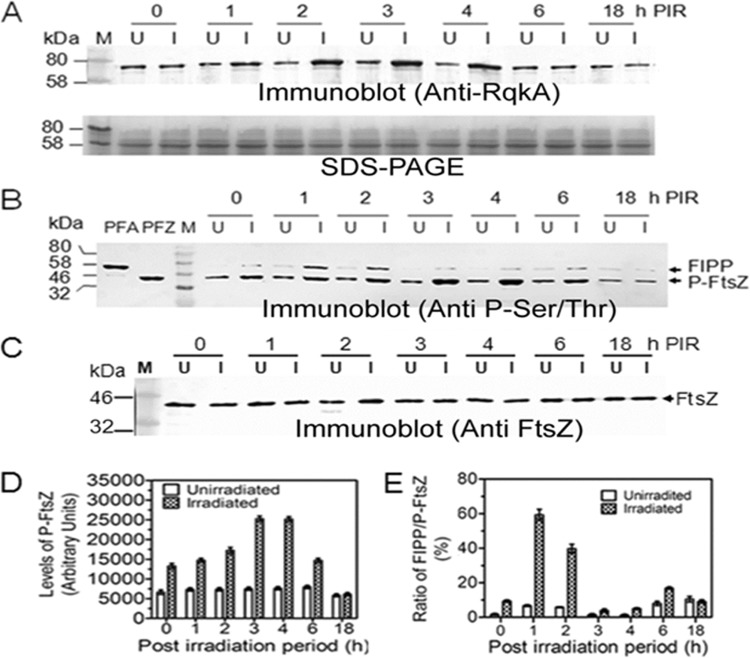
Phosphorylation status of FtsA and FtsZ in wild-type Deinococcus radiodurans. (A) Cells were grown to the exponential phase and irradiated to 6.0 kGy radiation. Gamma-irradiated cells (I) and the respective unirradiated (SHAM) control cells (U) were grown in fresh media, and aliquots were collected at different time points of postirradiation recovery (PIR). The cell lysate of wild-type cells expressing native RqkA was separated on SDS-PAGE and immunoblotted using antibodies against RqkA (Immunoblot Anti-RqkA) as detailed in Materials and Methods. (B and C) Similarly, wild-type cells were exposed to 6 kGy radiation, and aliquots were taken at different PIR times. Immunoprecipitation was done using FtsZ antibodies, and immunoprecipitates were analyzed for phosphoproteins (B) and total FtsZ (C) using antibodies against phospho-Ser/Thr epitope (Immunoblot Anti P-Ser/Thr) and FtsZ (Immunoblot Anti-FtsZ), respectively. The band intensities of P-FtsZ and FtsZ interacting phosphoprotein (FIPP) were estimated from panel B data using densitometric scanning. (D and E) Levels of P-FtsZ (D) and the ratio of FIPP to P-FtsZ (E) were determined and plotted using GraphPad Prism. Data in panel D and E represent means ± standard deviations (SD) (*n* = 3). Data given without statistical analysis are representative of results from the reproducible experiments repeated 3 times.

RqkA was induced in response to radiation exposure and showed a kinetic change during PIR (Fig. 2A). Since RqkA phosphorylates FtsZ *in vitro*, the kinetics of FtsZ phosphorylation was also monitored during PIR in D. radiodurans. In brief, total proteins from the cells collected at different PIR and their respective unirradiated controls, processed identically (SHAM controls), were subjected to immunoprecipitation (IP) using antibodies against FtsZ and phosphorylation in the precipitated components was checked using anti-phosphorylation Ser/Thr antibodies. Results showed that the phosphorylation of both FtsZ and an FtsZ interacting phosphoprotein (FIPP) changed kinetically during PIR. For instance, the levels of FtsZ phosphorylation were highest during h 3 and h 4 of PIR and then decreased to the levels seen at h 0 of PIR while they did not change in the SHAM controls (compare Fig. 2B and D). Interestingly, the FIPP was identified as the phosphorylated FtsA of D. radiodurans (Fig. S2). Notably, the levels of phosphorylation of FIPP (P-FtsA) increased in first 3 h PIR and then decreased almost to the levels seen with the unirradiated cells. This was also evident from the low ratio of P-FtsA to P-FtsZ at h 3 and h 4 of PIR (Fig. 2B and E). It may be noted that h 4 of PIR is the time point at which cells irradiated at 6 kGy resume growth and the RqkA level is found to be highest. The phosphorylation status of FtsA and FtsZ and the effect of in *trans* expression seen with these proteins as well as RqkA in D. radiodurans were further studied in wild-type cells expressing histidine-tagged FtsZ (His-FtsZ), FtsA (His-FtsA), and RqkA in *trans*. The expression of these recombinant proteins was confirmed by immunoblotting using polyhistidine antibodies or RqkA antibodies as required (data not given). The effect of in *trans* expression of His-FtsZ, His-FtsA, and RqkA on the growth characteristics of wild-type cells was monitored spectrophotometrically at 600 nm and by estimating CFU levels per milliliter. Interestingly, overexpression of RqkA had negatively impacted the growth of D. radiodurans compared to the untransformed wild type (Fig. S3). The levels of phosphorylation of FtsZ and FtsA in D. radiodurans were monitored under both normal and radiation stress growth conditions. It was observed that the FtsZ and FtsA proteins were phosphorylated under normal growth conditions and that the levels of the proteins had increased severalfold upon radiation exposure (Fig. 2D and E). Interestingly, we noticed that immunoprecipitates of the total proteins from the cells expressing His-FtsZ or His-FtsA showed induced phosphorylation by RqkA upon gamma radiation exposure and coprecipitation of the additional band corresponding to the size of native FtsA and FtsZ, respectively. These proteins were found to have hybridized with antibodies against phospho-Ser/Thr epitopes (Fig. S4). These results suggested a correlation between radiation-inducible expression of RqkA and the enhancement of FtsZ and FtsA phosphorylation *in vivo* on the one hand and subsequent alteration of the interactions between FtsZ and FtsA on the other.

10.1128/mSphere.00325-18.3 FIG S3  Growth characteristics of different derivatives of Deinococcus radiodurans. D. radiodurans R1 wild-type (WT) cells were independently transformed with plasmid expressing hexahistidine-tagged FtsA (HisFtsA) and FtsZ (HisFtsZ) and C18-tagged FtsA (FtsA-C18) as well as RqkA. The effect of their *trans* expression on cell division and growth was monitored under normal growth conditions by measuring levels of CFU per milliliter (A) and the optical densities in microtiter plates at 600 nm (B), respectively. Data shown represent averages of results from 6 replicates with SD. Download FIG S3, DOC file, 0.3 MB. Copyright © 2018 Maurya et al. 2018 Maurya et al. This content is distributed under the terms of the Creative Commons Attribution 4.0 International license.

10.1128/mSphere.00325-18.4 FIG S4  Effect of phosphorylation on FtsZ and FtsA interaction in Deinococcus radiodurans. Cell-free extracts of the D. radiodurans cells expressing histidine-tagged FtsA (HisFtsA) and FtsZ (HisFtsZ) on a low-copy-number plasmid were immunoprecipitated with antibodies against polyhistidine. Immunoprecipitates were separated on SDS-PAGE, blotted on a membrane, and hybridized with antibodies against histidine (A) and with phospho-Ser/Thr antibodies (B). Similarly, proteins from the wild-type cells harboring vectors were immunoprecipitated with antibodies against RqkA (RqkA) and histidine (Vector) and were also blotted with phospho-Ser/Thr antibodies (B). Download FIG S4, DOCX file, 1.4 MB. Copyright © 2018 Maurya et al. 2018 Maurya et al. This content is distributed under the terms of the Creative Commons Attribution 4.0 International license.

### FtsZ interaction with FtsA was affected in Escherichia coli cells expressing RqkA in *trans*.

**FIG 3:**
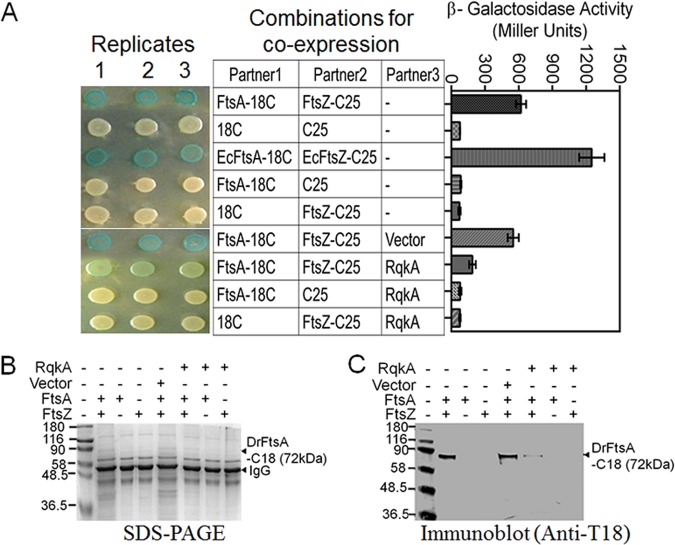
Effect of phosphorylation on *in vivo* interaction of FtsZ with FtsA in a surrogate Escherichia coli host. Translation fusions of FtsA and FtsZ were generated with T18 (FtsA-C18) and T25 (FtsZ-C25) tags of adenylate cyclase in BACTH plasmids, and their coexpression was ascertained by immunoblotting using antibodies against T18 and T25 tags, respectively (Fig. S3B to D). These plasmids were transformed into an E. coli BTH101 host expressing RqkA, FtsA, and FtsZ in different combinations. The interactions of these proteins were monitored as white-blue color colonies and as the expression of β-galactosidase in liquid culture (A). E. coli BTH101 harboring pVHS559 (Vector) and BACTH plasmids expressing T18 (C18) and T25 (C25) tags were used as negative controls. Cell-free extracts of E. coli BTH101 coexpressing FtsA-C18 and FtsZ-C25 in the presence and absence of RqkA were prepared and used for immunoprecipitation using T25 antibodies. Immunoprecipitates were separated on SDS-PAGE (B) and immunoblotted using antibodies against T18 domain of CyaA (Immunoblot anti-T18) (C) as detailed in Materials and Methods. Data in panel A are representative of results from a reproducible experiment repeated 3 times, while data in panels B and C are representative of results from a reproducible experiment repeated 2 times. β-Galactosidase activity data given in panel A represent means ± SD (*n* = 9).

Since FtsA is an interacting partner of FtsZ, which tethers the FtsZ ring to the membrane and stabilizes it (32), and since FtsZ and FtsA were found to be the phosphoproteins in D. radiodurans (Fig. 2B), we became curious about the status of the FtsA interaction with FtsZ in E. coli cells expressing RqkA as well as in the host bacterium. We examined the effect of FtsZ phosphorylation on its interaction with FtsA using a bacterial two-hybrid (BACTH) system and by coimmunoprecipitation (Co-IP) in recombinant E. coli. For BACTH studies, E. coli BTH101 cells expressing RqkA were transformed with plasmids coexpressing FtsA-C18 and FtsZ-C25 in different combinations. The expression of these proteins on plasmids was confirmed (Fig. S5). These cells were monitored for potential interactions that would lead to CyaA activity and hence to expression of β-galactosidase. It was observed that the FtsZ interaction with FtsA was reduced in the presence of RqkA (Fig. 3A). Results obtained from co-IP also supported this observation. E. coli cells coexpressing FtsA-C18 and FtsZ-C25 showed coprecipitation of FtsA-C18 with FtsZ only when RqkA was absent (Fig. 3B and C). However, in the presence of RqkA, the FtsZ showed a weak interaction with FtsA and the amount of FtsA (as FtsA-C18) was lower than that observed in the absence of RqkA (Fig. 3C). These results supported our previous observations indicating that RqkA can phosphorylate FtsZ, which could affect its interaction with FtsA in surrogate E. coli cells.

10.1128/mSphere.00325-18.5 FIG S5  Protein purification and expression analysis of BTH cell fusions and RqkA kinase in E. coli BTH101. Recombinant FtsZ, P-FtsZ, FtsA, and RqkA were purified and analyzed on SDS-PAGE (A). E. coli BTH101 was transformed with pVHSRqk, and expression levels of recombinant RqkA in recombinant E. coli (E. coli BTHRQK) were ascertained using antibodies against RqkA (B). E. coli BTHRQK was transformed with pUT18FtsA, expression levels of FtsA-C18 fusions were ascertained using antibodies against C18 tag, and the resultant E. coli strain was named E. coli BTHRQFTSA (C). E. coli BTHRQFTSA was transformed with pKNTFtsZ, and expression levels of FtsZ were confirmed using antibodies against the C25 tag of the BACTH system (D). Loading control of proteins used in SPR experiments (E). The sizes of the fusions were confirmed using molecular size markers (M). Download FIG S5, DOC file, 0.1 MB. Copyright © 2018 Maurya et al. 2018 Maurya et al. This content is distributed under the terms of the Creative Commons Attribution 4.0 International license.

### The FtsA interaction with FtsZ changes during PIR in wild-type Deinococcus radiodurans.

**FIG 4:**
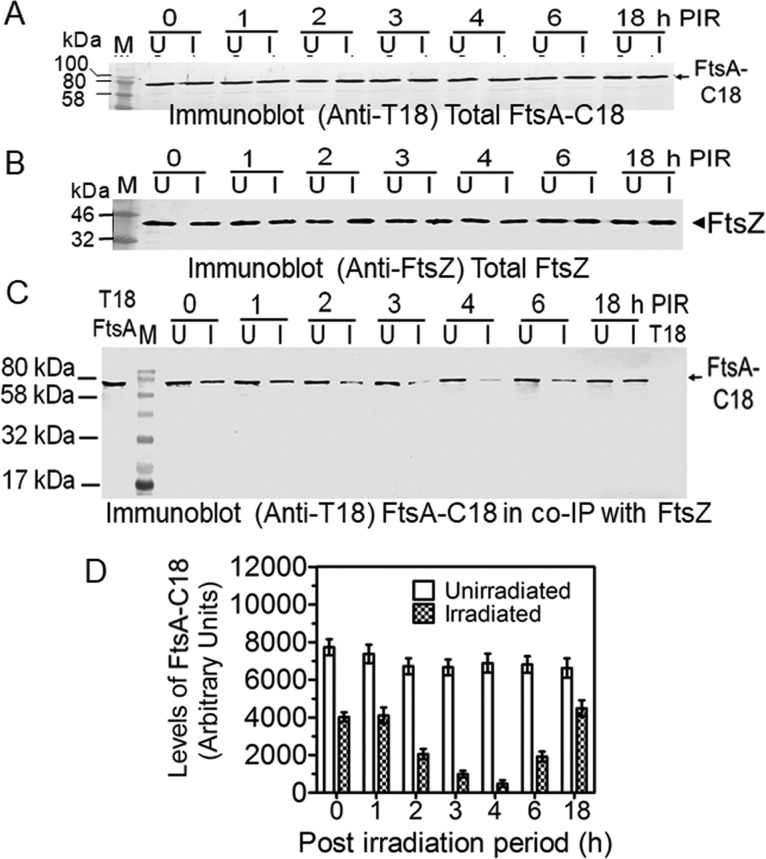
*In vivo* interaction of FtsA with FtsZ during postirradiation recovery in Deinococcus radiodurans. D. radiodurans cells expressing T18-tagged FtsA (FtsA-C18) were irradiated with 6.0 kGy radiation. Cells recovering from radiation stress (I) and the corresponding SHAM control cells (U) were collected at different time intervals, and cell-free extracts were prepared. The effect of phosphorylation and radiation on the interaction of FtsA with FtsZ was studied in the D. radiodurans cells expressing FtsA-C18 along with their native FtsZ as described in Materials and Methods. The levels of total FtsA-C18 (A) and total FtsZ (B) were measured using antibodies against T18 (Immunoblot anti-T18) and FtsZ (Immunoblot anti-FtsZ), respectively. Total proteins with nearly the same levels of FtsA-C18 and FtsZ were immunoprecipitated using deinococcal FtsZ antibodies. Immunoprecipitates were separated on SDS-PAGE and immunoblotted using anti-T18 antibodies (C), and the intensities of immunosignals were measured densitometrically and plotted (D). Data shown in blots are representative of results from the reproducible experiments repeated at least 3 times. Data given in panel D represent means ± SD (*n* = 6).

Since radiation treatment was found to be able to induce the phosphorylation of both FtsZ and FtsA concurrent with changes in the levels of RqkA (Fig. 2), we decided to study the kinetics of FtsZ and FtsA interactions during PIR in D. radiodurans. To that end, FtsA was fused with the T18 domain of CyaA and expressed in D. radiodurans. The interaction of FtsZ with FtsA was monitored by immunoprecipitation using antibodies against FtsZ of D. radiodurans followed by detection of FtsA (as FtsA-C18) using T18 antibodies. These cells expressing nearly identical levels of FtsA-C18 (Fig. 4A) and FtsZ (Fig. 4B) showed a kinetic change in the levels of FtsA-C18 associated with FtsZ during PIR (compare lane 1 in Fig. 4C to data in panel D). For instance, the amount of immunoprecipitated FtsA-C18 was very high in unirradiated cells as well as in cells in early PIR but was reduced significantly at h 3 and h 4 PIR in cells that apparently had higher levels of phosphorylated FtsZ (Fig. 2). On the other hand, the amount of FtsA-C18 that precipitated with FtsZ did not change in the unirradiated controls of all PIR samples (compare lanes U in Fig. 4C to data in panel D). Although the levels of total FtsA could not monitored due to nonavailability of antibodies against deinococcal FtsA, the quantitative real-time PCR data showed that the levels of FtsA did not change during PIR (Fig. S6). Thus, the kinetic changes in the amounts of P-FtsA (FIPP) (Fig. 2B) as well as FtsA (Fig. 4C) that were precipitated along with FtsZ were found to be nearly the same during PIR in D. radiodurans. For instance, both P-FtsA and FtsA in FtsZ immunoprecipitates were present at higher levels in h 1 and h 2 PIR samples than in h 3 and h 4 PIR samples. Since the co-IP was done using FtsZ antibodies in both the cases (Fig. 2B and 4C), these results together suggest that FtsA interaction with FtsZ seems to be largely controlled by FtsZ phosphorylation in D. radiodurans, and the possibility of reduced interaction of these proteins, if contributing to the cell cycle arrest in response to DNA damage, cannot be ruled out in this bacterium.

10.1128/mSphere.00325-18.6 FIG S6  The *ftsA* expression profile during postirradiation recovery in Deinococcus radiodurans. The total RNA prepared from the cells collected at different postirradiation recovery (PIR) periods was converted into cDNA. The quantitative real-time PCR was carried out using ftsA (drFtsA)-specific primers. Yields of PCR products were estimated using a standard curve prepared with a known amount of target DNA (top). There was no significant change in the levels of *ftsA* transcription during PIR (bottom). Download FIG S6, DOCX file, 0.03 MB. Copyright © 2018 Maurya et al. 2018 Maurya et al. This content is distributed under the terms of the Creative Commons Attribution 4.0 International license.

### The *rqkA* mutant lacks radiation effects on FtsZ phosphorylation and its interaction with FtsA.

**FIG 5:**
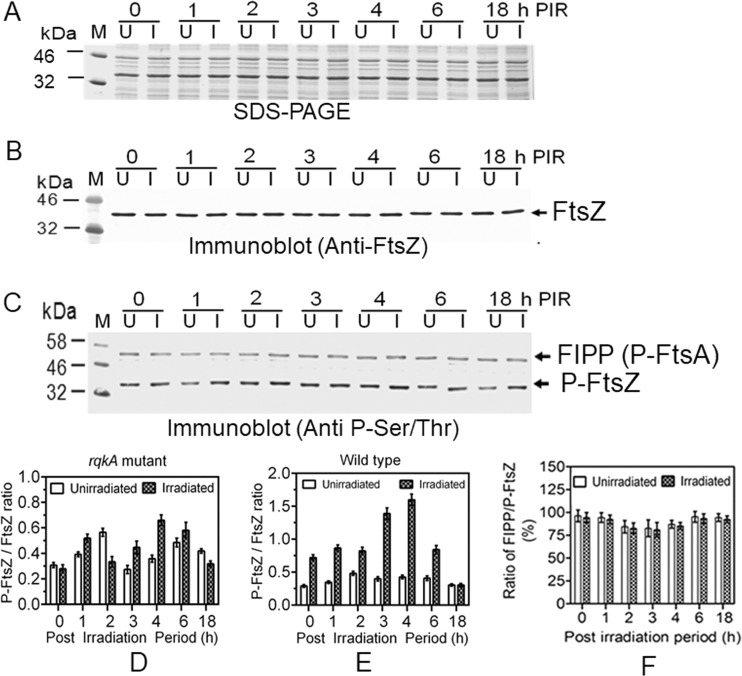
Phosphorylation status of FtsZ and FtsA in *rqkA* mutant of Deinococcus radiodurans. Cells were grown to the exponential phase and irradiated with 6.0 kGy radiation. Gamma-irradiated (I) and SHAM (unirradiated) control (U) cells were grown in fresh media, and the cells were collected at different time points of PIR. The cell lysates of *rqkA* mutant were separated on SDS-PAGE (A) and immunoblotted using FtsZ antibodies (Immunoblot anti-FtsZ) as detailed in Materials and Methods (B). Similarly, these samples were immunoprecipitated using FtsZ antibodies and the presence of a phosphoprotein(s) was detected in immunoprecipitates using phospho-Ser/Thr epitope antibodies (Immunoblot Anti P-Ser/Thr) (C). Band intensities of P-FtsZ and FtsZ were quantified densitometrically. The ratios of P-FtsZ to FtsZ for the *rqkA* mutant obtained from Fig. 2C data (D) and for the wild type from Fig. 2B data (E) were calculated and plotted as the means of arbitrary units with standard deviations using GraphPad Prism. Similarly, band intensities of P-FtsZ as well as FIPP were estimated densitometrically and ratios of FIPP to P-FtsZ were plotted (F). Data in panels D, E, and F represent means ± SD (*n* = 3), and the data presented without statistical analysis are representative of results from the reproducible experiments repeated 3 times.

The results obtained from *in vitro* and *in vivo* studies have clearly indicated that FtsZ and FtsA phosphorylation and the levels of RqkA change kinetically during PIR and that phosphorylation of FtsZ affects the interaction of FtsA with FtsZ. STPKs are known for promiscuity, and the regulation of specificity, if any, is not fully understood. When the levels of FtsZ and its phosphorylation were investigated in the *rqkA* mutant grown under normal and irradiated conditions (Fig. 5A), the FtsZ levels did not change during PIR of the mutant (Fig. 5B). Surprisingly, phosphorylation of FtsZ was observed, albeit at a low level compared to the wild type (Fig. 5C). But FtsZ phosphorylation was not radiation inducible and did not show a kinetic change during PIR in the absence of RqkA (Fig. 5C). Notably, the amount of FtsZ interacting phospho-protein (FIPP), which had been identified as P-FtsA (Fig. 2B), that coprecipitated with FtsZ also did not change during PIR in the *rqkA* mutant. For instance, the ratios of P-FtsZ to FtsZ were nearly the same in all the gamma-irradiated samples of the *rqkA* mutant (Fig. 5D), results which differed from the results seen with the corresponding samples of wild-type cells (Fig. 5E). Unlike the results seen with the wild type (Fig. 2D), the ratio of FIPP (P-FtsA) to P-FtsZ also did not change during PIR in the *rqkA* mutant (Fig. 5F). These results clearly suggested that although FtsZ becomes phosphorylated in this bacterium, the ¥ radiation stimulation of FtsZ phosphorylation seems to be contributed by RqkA, which is characterized as a DNA damage-responsive Ser/Thr protein kinase in this bacterium.

### FtsA and P-FtsA interactions with FtsZ and P-FtsZ show similar patterns *in vitro*.

**FIG 6:**
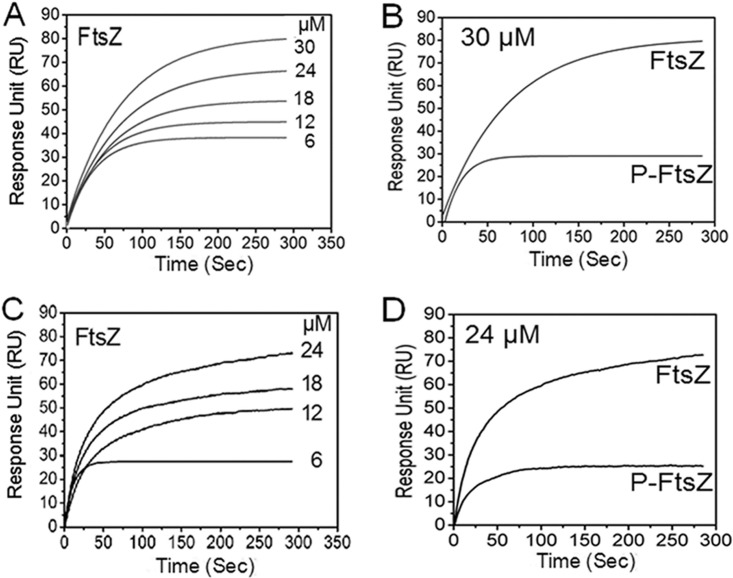
Effect of FtsA phosphorylation on its interaction with FtsZ and P-FtsZ using surface plasmon resonance. FtsA (25 µM) (A and B) or P-FtsA (25 µM) (C and D) protein was immobilized on a gold sensor chip followed by incubation with different concentrations (6 to 30 µM) of FtsZ (A and C) and its P-FtsZ phosphorylated form (data not shown) in the mobile phase. Surface plasmon resonance (SPR) signals were recorded and data processed as described in Materials and Methods. Comparative data showing the SPR signals with the highest concentrations of FtsZ and P-FtsZ incubated with FtsA (B) and P-FtsA (D) are shown. Data given are representative of results from the experiments repeated three times.

**FIG 7:**
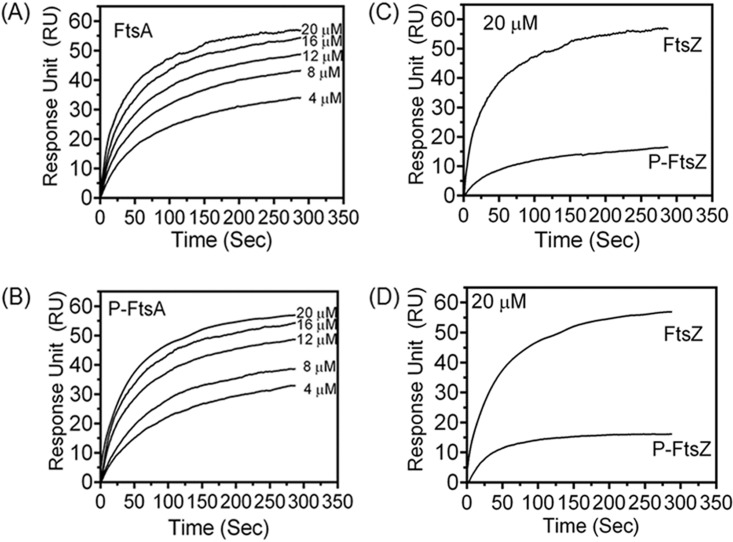
Effect of FtsZ phosphorylation on its interaction with FtsA and P-FtsA using surface plasmon resonance. FtsZ (20 µM) (A and B) or P-FtsZ (20 µM) (C and D) protein was immobilized on a gold sensor chip followed by incubation with different concentrations (4 to 20 µM) of FtsA (A and C) and its P-FtsA phosphorylated form (B and D) (data not shown for all concentrations except the highest concentration of FtsA or P-FtsA mobilized over P-FtsZ) in the mobile phase. Surface plasmon resonance (SPR) signals were recorded and data were processed using inbuilt kinetic evaluation software as described in Materials and Methods. Comparative data representing SPR signals with the highest concentrations of FtsA and P-FtsA incubated with FtsZ (C) and P-FtsZ (D) are shown. Data given are representative of results from the experiments repeated two times.

**FIG 8:**
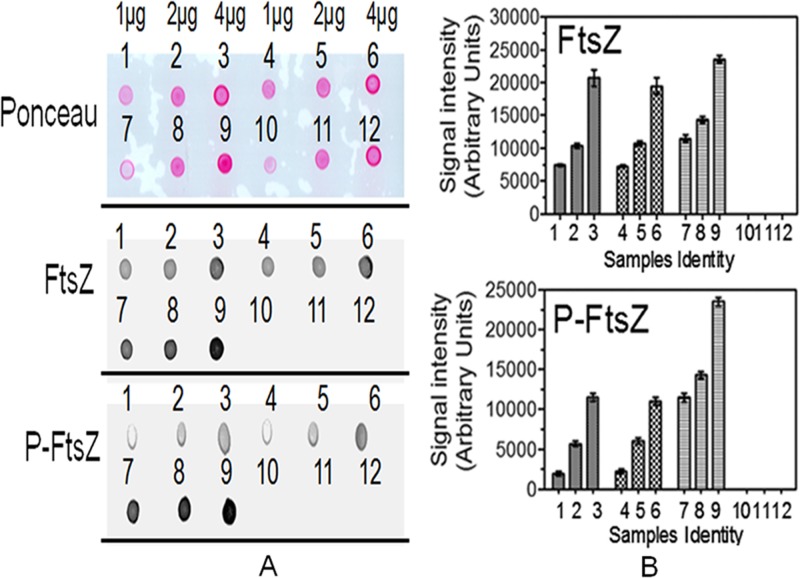
Effect of FtsA phosphorylation on its interaction with FtsZ and P-FtsZ using protein-protein hybridization. For determination of the data, different amounts (1 µg, 2 µg, and 4 µg) of FtsA (spots 1 to 3), P-FtsA (spots 4 to 6), FtsZ as the positive control (spots 7 to 9), and BSA as the negative control (spots 10 to 12) were spotted on nitrocellulose membranes in 3 replicates. One set was stained with Ponceau, and others were hybridized with FtsZ and P-FtsZ proteins (see Materials and Methods). Hybridization of FtsZ and P-FtsZ with target proteins was detected by immunoblotting using anti-FtsZ antibodies (A). Spot intensity was quantified densitometrically and plotted (B). Data given in panel B represent means ± SD (*n* = 3), and those without statistical analysis are representative of data from the experiments repeated three times.

The possibility of FtsA phosphorylation affecting its interaction with FtsZ was checked by surface plasmon resonance (SPR) and protein-protein hybridization studies, as described in Materials and Methods. FtsA and P-FtsA were immobilized independently on a bare gold sensor chip, and purified FtsZ and P-FtsZ (Fig. S5E) were allowed to pass over it in a mobile phase. The interaction of FtsA with FtsZ was confirmed by a concentration-dependent increase in the SPR signal, and nearly identical trends were seen with FtsA and P-FtsA (Fig. 6A and B). P-FtsZ did not interact efficiently with either of the forms of FtsA (Fig. 6C and D). The SPR analyses of these proteins were also carried out in a reverse manner by immobilizing FtsZ and P-FtsZ on a bare gold sensor chip and running FtsA and P-FtsA in the mobile phase, and the results were in good agreement (Fig. 7). The dissociation constant (*K*_*d*_) values were calculated for FtsZ, and data from the P-FtsZ interaction with FtsA and P-FtsA proteins as analyzed for the interactions shown in Fig. 6 and 7 are given in Table 1. These results indicated that the FtsA and P-FtsA interactions with FtsZ or P-FtsZ proteins were nearly identical. Interestingly, when we compared the association and dissociation equilibrium data of these interactions with the control results, we noticed that the FtsA/P-FtsA interaction with FtsZ/P-FtsZ showed a strong association but that the dissociation kinetics was consistently slow. On the other hand, the positive control showed a very good association and very good dissociation kinetics (Fig. S7). Results obtained from protein-protein hybridization studies also supported the SPR results with respect to the FtsA and FtsZ interactions. For instance, when FtsA and P-FtsA were immobilized on a nitrocellulose membrane and hybridized with FtsZ or P-FtsZ, we observed nearly identical levels of interaction of FtsZ and P-FtsZ, as detected using polyclonal antibodies against FtsZ, with both FtsA and P-FtsA (Fig. 8). These results together suggested that, at least *in vitro*, phosphorylation of FtsA is less likely to have any role in its interaction with FtsZ or P-FtsZ. Since FtsA is phosphorylated and since the P-FtsZ/P-FtsA ratio varies during PIR, the possibility of P-FtsA differentially regulating interactions of other divisome proteins during FtsZ ring formation and cytokinesis cannot be ruled out.

10.1128/mSphere.00325-18.7 FIG S7  The SPR curve of the positive control analyzed with different combinations of FtsA and FtsZ. The background signal of buffer was subtracted from specific SPR signals; a differential plot showing concentration-dependent protein-protein interactions is given. Download FIG S7, DOCX file, 0.2 MB. Copyright © 2018 Maurya et al. 2018 Maurya et al. This content is distributed under the terms of the Creative Commons Attribution 4.0 International license.

**TABLE 1:** Table for comparison of dissociation constants between FtsA and FtsZ variants in different combinations analyzed through SPR

Interacting partners		*K*_*d*_ (M)	Interaction level
Immobilized on gold	Mobile		
FtsZ	FtsA	7.93 × 10^−6^ ± 1.02 × 10^−7^	Strong
FtsZ	P-FtsA	5.17 × 10^−6^ ± 3.37 × 10^−7^	Strong
P-FtsZ	FtsA	6.52 × 10^−5^ ± 2.11 × 10^−6^	Weak
P-FtsZ	P-FtsA	5.83 × 10^−5^ ± 1.91 × 10^−6^	Weak
FtsA	FtsZ	6.83 × 10^−6^ ± 1.64 × 10^−7^	Strong
P-FtsA	FtsZ	8.35 × 10^−6^ ± 2.32 × 10^−7^	Strong
FtsA	P-FtsZ	1.03 × 10^−5^ ± 3.81 × 10^−6^	Weak
P-FtsA	P-FtsZ	1.50 × 10^−5^ ± 5.07 × 10^−6^	Weak

### FtsA failed to stimulate the GTPase activity of P-FtsZ.

**FIG 9:**
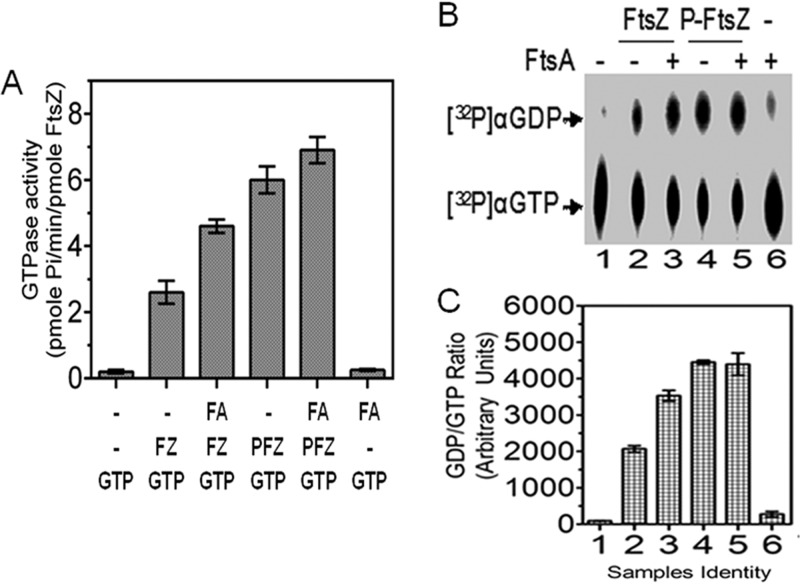
Effect of RqkA phosphorylation on the stimulatory effect of FtsA on GTPase activity of FtsZ. In brief, 5 µM purified recombinant FtsZ (FZ) and its phosphorylated form (PFZ) were checked for GTPase activity by malachite green assay (see Materials and Methods) in the presence and absence of 5 µM FtsA in different combinations. Release of inorganic phosphate was measured and computed as the GTPase activity (A). Data given represent means ± SEM (*n* = 3) of results from a reproducible experiment repeated 3 times. The stimulatory effect of FtsA on the GTPase activity of FtsZ and P-FtsZ was also checked by thin-layer chromatography (TLC) (B). In brief, 5 µM purified recombinant FtsZ and 5 µM of its phosphorylated form were incubated with 30 nM [α-^32^P]GTP substrate in the absence and presence of 5 µM purified FtsA in different combinations for 10 min (see Materials and Methods). [α-^32^P]GTP hydrolysis to [α-^32^P]GDP in different samples (samples 1 to 6) was monitored using TLC, and autoradiograms were developed. Band intensities of 1 to 6 corresponding to panel B were determined densitometrically, and GDP/GTP ratios (arbitrary units) were calculated and plotted as histograms using GraphPad Prism 5.0 (C). Data given in panel C represent means ± SD (*n* = 3).

FtsA is an important constituent of the Z ring, and its interaction with FtsZ and its stimulation of GTPase activity were demonstrated earlier (30, 31). Therefore, the possibility that FtsZ phosphorylation affects its GTPase activity, and its interaction with FtsA, was hypothesized. GTPase activity was monitored as the conversion of GTP to GDP and measured colorimetrically as well as chromatographically. FtsA stimulated the GTPase activity of FtsZ by nearly 1.5-fold, which was in agreement with the reported results. The level of inorganic phosphate (P_i_) released from GTP was nearly 2-fold higher in the case of P-FtsZ than in the case of FtsZ (Fig. 9A). However, FtsA did not stimulate the GTPase activity of P-FtsZ and the levels were nearly identical irrespective of the presence or absence of FtsA in the case of P-FtsZ. When GTP hydrolysis was measured as the conversion of [^32^P]GTP to [^32^P]GDP in the presence and absence of FtsA in different combinations, similar results were observed (Fig. 9B and C). For instance, P-FtsZ showed significantly higher GTPase activities than wild-type FtsZ in the absence of FtsA (Fig. 9A and B; compare lane 2 with lane 4). However, in the presence of FtsA, the GTPase activity of P-FtsZ was not stimulated compared with wild-type FtsZ (Fig. 9A and B; compare lane 2 with lane 3 and lane 4 with lane 5). Phosphorylated FtsA produced effects on GTPase activity stimulation of FtsZ nearly identical to those seen with the unphosphorylated form (data not shown). Such a loss of FtsA stimulation of GTPase activity in the case of P-FtsZ could be accounted for the loss of the interaction between these proteins.

## DISCUSSION

Cell cycle regulation in response to DNA damage is one of the most elegantly studied processes in eukaryotes. It involves cascades of protein phosphorylation/dephosphorylation by differential regulation of eSTPKs and tyrosine kinases and their respective phosphatases. In bacteria, the regulation of cell division and of DNA repair (synonymous with the cell cycle in eukaryotes) mainly works through the SOS response mechanism, where a large number of proteins are induced in response to DNA damage. Some of these proteins take part in DNA repair, while some restrict cell division until DNA repair is completed (33). SOS response-mediated bacterial cell division arrest involves attenuation (direct or indirect) of FtsZ functions, mainly, GTPase activity, localization, and polymerization/depolymerization dynamics. Although it is known that FtsZ function is regulated by protein-protein interactions in the divisome, except for a few components, the modes of action of many divisome proteins or the associated regulators are not explicitly understood. How can SOS proteins inhibit FtsZ functions when several activators are also present in the cell? What determines the relative affinities of activators and inhibitors for FtsZ? Phosphorylation/dephosphorylation-based regulation could be one such mechanism that can explain the differential affinity of activators and inhibitors for FtsZ. The idea of the involvement of S/T phosphorylation in the regulation of bacterial cell cycle has been supported recently (15, 16). Further, the phosphorylation of cell division proteins such as FtsZ and DivIVA and their involvement in the regulation of cell division and genome segregation have also been shown in other bacteria under stress conditions (27, 34–36). Phosphorylation of DNA repair proteins such as single-stranded DNA-binding protein (SSB) and RecA has been also reported in B. subtilis, and an effect of phosphorylation on stimulation of these protein functions has been shown previously (37, 38). More recently, the involvement of ParB phosphorylation in the regulation of functions of ParABS, a segrosome complex, in genome segregation of M. tuberculosis has been shown (39).

D. radiodurans is a highly radioresistant bacterium that shows growth arrest in response to DNA damage (30). The classical LexA/RecA-type SOS response mechanism and the homologues of known SOS proteins that attenuate FtsZ function in other bacteria are absent in D. radiodurans (23, 24). Therefore, a possibility of an alternate mechanism(s) of cell cycle regulation cannot be ruled out in D. radiodurans. Recently, the involvement of S/T phosphorylation in the regulation of DNA repair proteins has been shown in this bacterium. It was found that the phosphorylation of two DNA repair proteins like PprA and D. radiodurans RecA (DrRecA) by RqkA significantly improved the activities of these proteins and their roles in radioresistance (28, 29). Since we observed that RqkA could also phosphorylate a number of important cell division proteins, including FtsZ and FtsA (see Fig. S8 in the supplemental material), and since the level of FtsZ does not change during PIR (30), the involvement of phosphorylation in the regulation of FtsZ function and its interaction with divisome partners such as FtsA were investigated. Here, we have brought forth strong evidence to suggest that both FtsZ and FtsA undergo phosphorylation in D. radiodurans and that FtsZ phosphorylation plays an important role in the regulation of the FtsZ interaction with FtsA. We demonstrated that FtsZ phosphorylation had severely affected its interaction with FtsA as measured in E. coli as well as in D. radiodurans, and the GTPase activity of FtsZ was stimulated. Further, it has been observed under both *in vitro* and *in vivo* conditions that the interaction of FtsZ and P-FtsZ does not change when FtsA is phosphorylated. Interestingly, we observed that, unlike E. coli and Bacillus subtilis, in the case of D. radiodurans the localization of FtsZ-green fluorescent protein (FtsZ-GFP) was not affected by gamma radiation but the FtsZ dynamics was arrested (Fig. S9). We observed three types of populations: those forming foci at juxtaposed positions in the membrane ("separating foci"), those whose foci progressed from the membrane ("approaching foci"), and those whose foci collapsed in the center ("closed foci"). We observed that the cells grown under normal conditions showed larger fluctuation in the number of cells forming either closed foci or approaching foci during PIR, which upon radiation exposure become nearly constant for 4 h PIR and then start showing patterns similar to those seen with untreated cells. We previously showed that cells exposed to gamma radiation were arrested for growth and that the growth resumed after ~4 h PIR (30). How phosphorylation of FtsZ can arrest FtsZ dynamics *in vivo* is not clear. However, the possibility of higher GTPase activity in P-FtsZ resulting in a higher rate of depolymerization (and in the absence of the FtsA interaction, if this leads to an impairment of FtsZ ring formation) cannot be ruled out. Earlier real-time imaging studies showed that FtsA plays a dual function in FtsZ dynamics (40). Those authors showed that it initially recruits the highly dynamic FtsZ polymers to the membrane and supports polymerization and that it causes destabilization of FtsZ polymer at a later stage. Thus, it seems that the phosphorylation of these proteins by a DNA damage-responsive protein kinase (RqkA) could inhibit their interactions, which can arrest cell division progression until the damaged DNA is repaired. Once the cells have recovered from DNA damage and these proteins are dephosphorylated by an as-yet-unknown mechanism, they can interact normally and cell division can commence. Changes in the phosphorylation of FtsZ and FtsA during PIR and its effect on the interaction of these proteins supported the proposition of a possible arrest in FtsZ dynamics during the post-DNA damage and repair phase in this bacterium, which would be studied independently. The effect of phosphorylation in the regulation of FtsZ activity and its interaction with FipA has been demonstrated in M. tuberculosis (35).

10.1128/mSphere.00325-18.8 FIG S8  RqkA phosphorylation of cell division proteins of D. radiodurans. A 0.5-µg to 1-µg volume of cell division proteins such as FtsZ, FtsK, FtsA, MinC, MinD, and DivIVA was incubated in the absence and presence of a 25×-lower molar concentration of RqkA and 30 nM of [γ-^32^P]ATP, and mixtures were separated on SDS-PAGE. The gels were dried, and an autoradiogram was developed. Download FIG S8, DOCX file, 0.5 MB. Copyright © 2018 Maurya et al. 2018 Maurya et al. This content is distributed under the terms of the Creative Commons Attribution 4.0 International license.

10.1128/mSphere.00325-18.9 FIG S9  Effect of gamma radiation on localization and dynamics of FtsZ in Deinococcus radiodurans. Deinococcus radiodurans R1 expressing FtsZ-GFP on a low-copy-number plasmid in D. radiodurans was grown under normal conditions (unirradiated [UI]) and exposed to 6.5 kGy gamma radiation (Ir). FtsZ was localized in the whole-cell population. Nearly 100% of the cells showed GFP-FtsZ localization. The cells showing GFP foci at different positions were divided into the following 3 categories: (i) foci in cells at juxtaposed positions (called "separating foci" [SF]); (ii) juxtaposed foci that moved onto the septum (called "approaching foci" [AF]); (iii) foci that were fused at the middle position (called "closed foci" [CF]). Download FIG S9, PDF file, 0.2 MB. Copyright © 2018 Maurya et al. 2018 Maurya et al. This content is distributed under the terms of the Creative Commons Attribution 4.0 International license.

**FIG 10:**
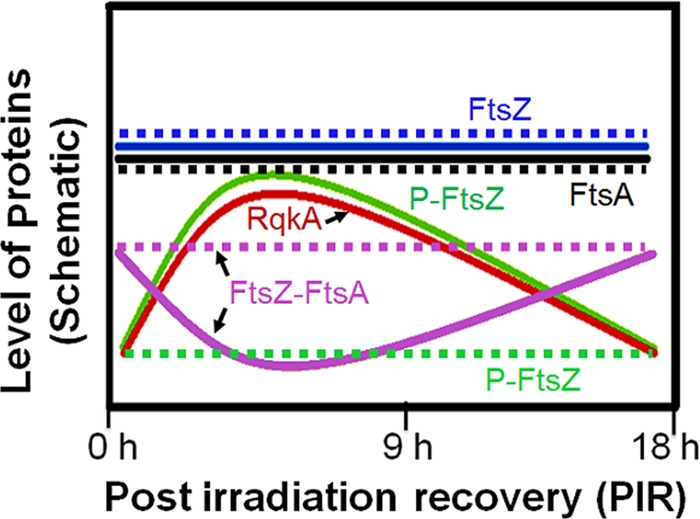
Schematic representation showing the changes in the levels of different proteins during postirradiation recovery. Changes in the levels of RqkA (red solid line), FtsA (black lines), and both the phosphorylated (green lines) and nonphosphorylated (blue lines) forms of FtsZ in both the wild-type strain (solid lines) and the *rqkA* mutant (dotted lines) are depicted based on the results shown elsewhere in the article. The pattern of interaction of FtsA and FtsZ (purple lines) during postirradiation recovery of *D. radiodurans* cells exposed to 6 kGy radiation is shown for the wild-type and *rqkA* mutant strains.

In summary, we show that RqkA, a DNA damage-responsive protein kinase of D. radiodurans, phosphorylates several important cell division proteins (Fig. S8), including FtsZ and FtsA of this bacterium. We had shown earlier that phosphorylation of DNA repair proteins such as PprA and RecA by RqkA improves their roles in the radioresistance of D. radiodurans. Here, we showed that in D. radiodurans, RqkA phosphorylates FtsZ and FtsA and phosphorylation of FtsZ affects the GTPase activity of FtsZ and its interaction with cognate FtsA *in vitro*. Further, these are found to be phosphoproteins in this bacterium, and their RqkA-mediated phosphorylation is radiation inducible and showed a kinetic change during PIR in the wild type (Fig. 10). Although FtsZ phosphorylation continued in the *rqkA* mutant background, three characteristic signatures of FtsZ during PIR ([i] radiation responsiveness, [ii] kinetics in phosphorylation, and [iii] FtsZ interaction with FtsA [in the form of FIPP]) were not observed. This strongly indicated that RqkA regulates FtsZ phosphorylation in response to radiation at the site(s) that seems to regulate FtsZ interaction with FtsA. Although studies on the mapping of phosphorylation sites in FtsZ and FtsA and further studies on the roles of such sites in the regulation of cell division would be required to emphasize the further role(s) of phosphorylation in cell cycle regulation (which would be done independently), the available evidence clearly supports the idea that FtsZ and FtsA are phosphoproteins and their phosphorylation is inducible by radiation in wild-type D. radiodurans. The lack of radiation inducibility in FtsZ and FtsA phosphorylation in cells lacking RqkA further emphasizes this kinase role in the radiation responsiveness of phosphorylation of these proteins. Thus, RqkA-mediated stimulation of the activity of DNA repair proteins (28, 29) and the role of RqkA in radiation-inducible phosphorylation of FtsZ and FtsA affecting functions of these proteins together provide strong support for the idea that RqkA is a cell cycle regulatory kinase in D. radiodurans. The reporting of a role of any DNA damage-responsive Ser/Thr protein kinase in bacterial cell cycle regulation would represent the first such report from this bacterium, which apparently lacks LexA/RecA-type classical SOS response-mediated cell cycle regulation.

## MATERIALS AND METHODS

### Bacterial strains, plasmids, and materials.

D. radiodurans R1 (ATCC 13939), a kind gift from J. Ortner, Germany (41), was grown in TGY medium (1% Bacto tryptone, 0.1% glucose, 0.5% yeast extract) with shaking at 180 rpm at 32°C. E. coli strain NovaBlue was used for cloning and maintenance of all the plasmids; E. coli strain BTH101 (lacking *cyaA*) (referred to here as BTH101) was used for coexpression of these proteins on BACTH plasmids for *in vivo* protein-protein interaction studies and was grown at 30°C for all experimental purposes. E. coli strain BL21(DE3) pLysS was used for expression of recombinant proteins. E. coli cells harboring pUT18, pKNT25, and pET28a(+) and its derivatives were maintained in the presence of the required antibiotics. Shuttle expression vector pVHS559 (42) and a derivative of p11559 (43) and its derivatives were maintained in the presence of spectinomycin (70 µg/ml) in E. coli and D. radiodurans. The pRADgro plasmid (44) and its derivatives were maintained in the presence of ampicillin (100 µg/ml) in E. coli and in the presence of chloramphenicol (8 µg/ml) in D. radiodurans. Standard protocols for all recombinant techniques were as described previously (45). All the bacterial strains and plasmids used in this study are listed in Table S1 in the supplemental material. Antibodies against the T18 (SC-13582) and T25 (SC-33620) domains of CyaA of Bordetella pertussis were procured commercially (Santa Cruz Biotechnology, Inc.). Antibodies against FtsZ and RqkA of D. radiodurans were commercially produced in rabbit (Merck, Millipore, India). Molecular-biology-grade chemicals and enzymes were procured from Sigma Chemicals Company, USA; Roche Biochemicals, Mannheim, Germany; New England Biolabs (USA); and Merck India Pvt. Ltd., India. Radiolabeled nucleotides were obtained from the Board of Radiation and Isotope Technology (BRIT), Department of Atomic Energy, India.

10.1128/mSphere.00325-18.10 TABLE S1  Lists of primers, bacterial strains, and plasmids used in this study. Download TABLE S1, DOC file, 0.1 MB. Copyright © 2018 Maurya et al. 2018 Maurya et al. This content is distributed under the terms of the Creative Commons Attribution 4.0 International license.

### Construction of recombinant plasmids.

Details of the strains, primers, and plasmids used in this study are given in Table S1. In brief, the coding sequences of RqkA (DR_2518) were PCR amplified using 2518F and 2518R primers and cloned at NdeI and XhoI sites in pVHS559, yielding pVHSRqk. The *ftsA* gene was cloned in pET21a(+) and pET28a+ at NdeI and BamHI sites, yielding p21FTSA and pFTSA, respectively. These plasmids were used for the purification of FtsA. The T18 tag translationally fused through the C terminus of the coding sequence of FtsA (referred to here as *ftsA*-C18) was PCR amplified from pUTDFA (31) using BTHF(PV) and BTHR(PV) primers and subcloned in pVHS559 at NdeI and XhoI sites to yield pVHSFtsA18. Coding sequences of polyhistidine-tagged FtsA and FtsZ were PCR amplified using pETHisFw and pETHisRw primers and their respective pET28a(+) clones as a template and were subcloned in plasmid pRADgro at ApaI and XbaI sites, yielding pRadHisFA and pRadHisFZ, respectively, for their expression in D. radiodurans. Expression of all fusion proteins was confirmed by Western blotting using antibodies against the T18 domain for fusions named with C18 and hexahistidine designations for polyhistidine-tagged proteins (data not shown). The growth kinetics of D. radiodurans expressing HisFtsA, FtsA-C18, HisFtsZ, and RqkA (from pVHSRqk) in *trans* along with that of the wild type was monitored in 24-well microtiter plates. Recombinant plasmids pFTSZ, pFTSA, pUTDFA, and pKNTFZ used in this study were constructed earlier and described previously (30, 31), while pET2518 expressing recombinant RqkA was also described previously (26).

### Purification of recombinant proteins.

Recombinant FtsZ, FtsA, and RqkA were expressed on pFTSZ, pFTSA, and pET2518 in E. coli BL21(DE3) pLysS, respectively. The recombinant proteins were purified from the respective transgenic cells as described earlier (30), while RqkA was purified as described previously (26). In brief, cultures of E. coli BL21(DE3) pLysS expressing recombinant proteins grown overnight were diluted 1:100 in fresh LB broth containing 25 µg/ml kanamycin, 0.5 mM isopropyl-β-d-thiogalactopyranoside (IPTG) was added at an optical density (OD) at 600 nm of 0.3, and cells were harvested after 3 h postinduction. The cell pellet was suspended in buffer A (20 mM Tris-HCl [pH 7.6], 150 mM NaCl) containing 10 mM imidazole, 0.5 mg/ml lysozyme, 1 mM phenylmethylsulfonyl fluoride (PMSF), 0.03% NP-40, 0.03% Triton X-100, and 10% glycerol and incubated at 37°C for 30 min. Protease inhibitor cocktail was added to the reaction mixture, and the cells were sonicated for 10 min using 30-s pulses with intermittent cooling for 1 min at 25% amplitude. The cell lysate was centrifuged at 11,000 rpm for 30 min at 4°C. The cell extract was loaded onto a NiCl_2_ charged-fast-flow-chelating Sepharose column (GE Healthcare) preequilibrated with buffer A. The column was washed with 20 column volumes of buffer A containing 50 mM imidazole until proteins stopped coming from the column. Recombinant proteins were eluted with buffer A containing 250 mM imidazole. Fractions were analyzed by SDS-PAGE, and those containing nearly pure proteins were pooled and repurified by the use of a nickel-nitrilotriacetic acid (Ni-NTA) agarose column following the protocols described by the manufacturer (Qiagen, Inc.). Proteins were eluted in steps using 100 mM, 200 mM, 250 mM, and 300 mM imidazole in buffer A and analyzed on 10% SDS-PAGE. Fractions containing more than 95% pure protein were pooled and concentrated using 10-kDa-cutoff spin columns and then centrifuged at 16,000 rpm for 60 min to remove aggregates. The supernatant containing mostly soluble protein was dialyzed in buffer A containing 10 mM Tris-HCl (pH 7.6), 50 mM KCl, 50% glycerol, and 1 mM PMSF and stored at −20°C. For purification of the phosphorylated form of FtsA and of FtsZ, their respective plasmids were cotransformed with RqkA-expressing plasmid pRAD2518 in the E. coli BL21 strain, induced with IPTG, and purified (along with phosphatase inhibitor in lysis buffer) as mentioned above. Phosphorylation was checked using anti-phospho-Ser/Thr epitope antibodies (Cell Signaling Technology, Inc., USA) and standard protocols. Protein concentrations were determined by the Bradford method.

### Protein-protein interaction studies using surrogate Escherichia coli cells.

*In vivo* interactions of different proteins were monitored using a bacterial two-hybrid system (BACTH) as described earlier (46–48). In brief, BTH101 was transformed with different plasmids expressing target proteins with T18 tags (referred to here as C18 and N18 for T18 present at the C terminus and N terminus of target proteins, respectively) or T25 tags (referred to here as C25 and N25 for T25 present at the C terminus and N terminus of target proteins, respectively) and RqkA was expressed on pVHSRqk. Empty vectors were transformed in different combination and used as controls. Recombinant cells were scored on LB agar plates supplemented with appropriate antibiotics. These cells were grown overnight in triplicate, and 5 µl of the reaction mixture was spotted on LB agar plates containing 5-bromo-4-chloro-3-indolyl-β-d-galactopyranoside (X-Gal) (40 µg/ml), IPTG (0.5 mM), and antibiotics as required. Plates were incubated at 30°C overnight, and the appearance of white-blue color colonies was recorded. In parallel, an aliquot of the same culture was grown overnight with 0.5 mM IPTG and appropriate antibiotics, and β-galactosidase activity was measured from liquid cultures as described earlier (22, 48). In brief, the cultures were diluted 1:4 into LB medium and optical density at 600 nm was normalized. Cultures (100 µl) were mixed with 1 µl Z-buffer (60 mM Na_2_HPO_4_, 40 mM NaH_2_PO_4_, 10 mM KCl, 10 mM MgSO_4_, 50 mM β-mercaptoethanol, pH 7.0). To this mixture, 0.01% SDS and 20 µl chloroform were added to permeabilize the cells and cell debris was removed. Enzyme activity was measured in triplicate with 50 µl of supernatant using 0.4% O-nitrophenyl-β-d-galactoside (ONPG) as a substrate as described recently (22). The β-galactosidase activity was calculated in Miller units as described earlier (48).

### Western blotting and coimmunoprecipitation.

Different derivatives of BACTH plasmids expressing FtsA-C18 and C25 fusions of FtsZ were cotransformed in different combinations into BTH101 cells harboring either pVHS559 plasmid (vector) or pVHSRqk plasmid (expressing RqkA). The recombinant cells coexpressing these proteins were induced with 0.5 mM IPTG, and cells were lysed in radioimmunoprecipitation assay (RIPA) buffer (50 mM Tris base, 150 mM NaCl, 5 mM EDTA) containing 0.5% Triton X-100, 1 mM PMSF, 1 mM dithiothreitol (DTT), 0.5 mg/ml lysozyme, and 50 µg of a protease inhibitor cocktail tablet followed by sonication. The clear cell-free extracts were immunoprecipitated using polyclonal antibodies against the T25 tag to precipitate FtsZ. Immunoprecipitates were separated on a 10% SDS-PAGE gel, blotted onto a polyvinylidene difluoride (PVDF) membrane, and hybridized with monoclonal antibodies against the T18 tag to detect FtsA-C18 if present with the FtsZ or in its phosphorylated form. Hybridization signals were detected using anti-mouse secondary antibodies conjugated with alkaline phosphatase using BCIP/NBT (5-bromo-4-chloro-3-indolylphosphate/nitroblue tetrazolium) substrates (Roche Biochemical, Mannheim, Germany). Similarly, coexpression of RqkA and fusion proteins in recombinant BTH101 cells was monitored using antibodies against RqkA as well as against T18 and T25 domains of adenylate cyclase as described earlier (22). In brief, the total proteins of E. coli BTH101 cells coexpressing these recombinant proteins were separated on a 10% SDS-PAGE gel, blotted on a PVDF membrane (Millipore), and hybridized using primary antibodies against each of these proteins separately. The hybridization signals were detected by the use of anti-mouse and anti-rabbit secondary antibodies conjugated with alkaline phosphatase using BCIP/NBT substrates (Roche Biochemical, Mannheim, Germany).

### *In vivo* protein-protein interaction studies in Deinococcus radiodurans.

Translation fusions of FtsZ were generated with a polyhistidine tag, and translational fusions of FtsA were generated with T18 (FtsA-C18) of CyaA (Table S1). The expression of each fusion protein in D. radiodurans was ascertained by immunoblotting using antibodies against the polyhistidine tag and the T18 tag, respectively. These plasmids were cotransformed into D. radiodurans R1 in different combinations. Cell-free extracts of D. radiodurans coexpressing FtsA-C18 with His-FtsZ along with their controls were prepared. In brief, deinococcal cells coexpressing different proteins were pelleted and washed with 70% ethanol followed by a wash with phosphate-buffered saline. Pellets were suspended in 500 µl of lysis buffer A (50 mM Tris [pH 7.5], 100 mM NaCl, 1 mM PMSF, 5 mM MgCl_2_, 1 mM dithiothreitol [DTT], 0.5% Triton X-100) with 0.5 mg/ml lysozyme and 50 µg protease inhibitor cocktail tablet (Roche Biochemicals) followed by sonication on ice. Cell debris was removed by centrifugation at 2,000 × *g* for 10 min at 4°C. The clear cell-free extracts were immunoprecipitated using 1 µg monoclonal antibodies against the polyhistidine tag by following the protocol specified for a protein G immunoprecipitation kit (Sigma-Aldrich). Immunoprecipitates were separated on a 10% SDS-PAGE gel, blotted onto a PVDF membrane, and hybridized with monoclonal antibodies against the T18 tag. Hybridization signals were detected using anti-mouse secondary antibodies conjugated with alkaline phosphatase using BCIP/NBT substrates (Roche Biochemical, Mannheim, Germany).

For analysis of *in vivo* interactions of FtsA and FtsZ during PIR in D. radiodurans cells, the cells expressing FtsA-C18 on the plasmid (pVHSFtsA18) were grown in TGY broth containing 5 mM IPTG and mid-log-phase cells were irradiated with 6 kGy gamma radiation at a dose rate of 1.875 kGy/h (GC5000; BRIT, Department of Atomic Energy, India) and allowed to recover in TGY medium containing 5 mM IPTG as described earlier (44). Different aliquots and corresponding SHAM controls were collected, washed with 70% ethanol, and snap-frozen in liquid nitrogen and were then stored at −70°C overnight. Frozen cells were thawed and suspended in 500 µl of lysis buffer A (50 mM Tris-HCl [pH 7.5], 100 mM NaCl, 1 mM PMSF, 5 mM MgCl_2_, 1 mM dithiothreitol [DTT], 0.5% Triton X-100) with 0.5 mg/ml lysozyme and a 50-µg protease inhibitor cocktail tablet (Roche Biochemicals) followed by sonication on ice. Cell debris was removed by centrifugation at 22,000 × *g* for 10 min at 4°C. The clear cell-free extracts were immunoprecipitated using anti-FtsZ antibody by following the kit protocol described in a protein G immunoprecipitation kit (Sigma Aldrich). Immunoprecipitates were separated on a 10% SDS-PAGE gel, blotted onto a PVDF membrane, and hybridized with monoclonal antibodies against the T18 tag. Hybridization signals were detected using anti-mouse secondary antibodies conjugated with alkaline phosphatase using BCIP/NBT substrates (Roche Biochemical, Mannheim, Germany). Band intensities were quantified by the use of ImageJ 2.0 software and plotted with standard deviations in GraphPad Prism5.

### Surface plasmon resonance and protein-protein hybridization studies.

Surface plasmon resonance (SPR; AutoLab Esprit, the Netherlands) was employed to study the interaction of FtsA and P-FtsA with FtsZ and P-FtsZ using modifications of methods used earlier (49, 50). FtsA protein or P-FtsA protein (25 µM) was immobilized on a bare gold sensor chip employing EDC [1-ethyl-3-(3-dimethylaminopropyl)-carbodiimide]-NHS (*N*-hydroxysuccinimide) chemistry (AutoLab Esprit user manual) at 20°C, resulting in about 200 response units, using running buffer (20 mM Tris [pH 7.6]). FtsZ and P-FtsZ were used in the mobile phase. Different concentrations (6 to 30 µM) of the FtsZ and P-FtsZ proteins were incubated with 1 mM GTP and 1 mM MgCl_2_ and injected onto the FtsA-bound or P-FtsA-bound sensor chip in one channel. Tris (20 mM) (pH 7.6) containing 1 mM GTP and 1 mM MgCl_2_ was used as a buffer control to flow from another channel over immobilized FtsA or P-FtsA. Another pair of proteins showing equilibrium in their association and dissociation kinetics was used as a positive interaction control. The buffer controls were deducted, and the data were processed using AutoLab kinetic evaluation software (V5.4) and plotted after curve smoothing performed using OriginPro 8 software.

For protein-protein hybridization studies, different amounts (1 µg, 2 µg, and 4 µg) of FtsA and P-FtsA were spotted on a nitrocellulose membrane in duplicate and allowed to air dry at room temperature for 2 h as described earlier (31). FtsZ and bovine serum albumin (BSA) were used as positive and negative controls, respectively. One set was stained with Ponceau red, and the other set was used for hybridization. For hybridization, the membrane was wetted in buffer B (50 mM Tris-Cl [pH 7.6], 150 mM NaCl, 3% glycerol, 0.05% IGEPAL [Sigma-Aldrich, USA]) and then incubated in blocking solution (buffer B containing 3% skimmed milk powder) for 1 h. Blots were washed with buffer B and resoaked overnight in 5 ml of buffer B containing 1 mg/ml of FtsZ or P-FtsZ and incubated on a shaker at 4°C. Blots were washed with buffer B and incubated for 2 h at room temperature in blocking solution containing FtsZ antibodies raised in rabbit at 1:50,000 dilutions as described earlier (31). The membrane was washed with buffer B and incubated for 2 h at room temperature in blocking solution containing anti-rabbit IgG conjugated with alkaline phosphatase. The membrane was washed extensively with buffer B and developed with NBT-BCIP substrate. Each dot was quantified using ImageJ 2.0 software and plotted by the use of GraphPad Prism5 software.

### Protein phosphorylation.

Protein phosphorylation studies were carried out as described earlier (26, 28). In brief, the purified proteins were mixed with the increasing concentrations of purified RqkA kinase in order to achieve substrate-to-kinase molar ratios of 1:1 to 100:1 in a buffer (70 mM Tris-HCl [pH 7], 5 mM DTT, 10 mM MgCl_2_) containing 100 µM ATP and 50 nM [γ-^32^P]ATP and incubated at 37°C for 1 h. For *in vitro* phosphorylation of cell division and DNA-binding proteins, target proteins (50 to 200 nM) were mixed with RqkA to achieve a substrate-to-kinase molar ratio of 25:1. For coexpression and detection of phosphorylated form of FtsA with FtsZ, plasmid p21FTSA expressing FtsA was cotransformed with FtsZ-expressing plasmid along with in *trans* expression of RqkA (from pVHSRqk) in E. coli BL21(DE3) pLysS cells. Expression of the recombinant proteins was induced with IPTG, and the total cell lysate was separated on SDS-PAGE. Proteins were transferred onto a PVDF membrane and probed with polyclonal phospho-Ser/Thr antibodies (Cell Signaling Technology, Inc., USA) as described earlier (29). The E. coli strain expressing RecA of D. radiodurans on pETrecA was used as a positive control (29).

### Western blotting and *in vivo* phosphorylation from Deinococcus radiodurans.

D. radiodurans cells expressing FtsA-C18 were irradiated with 6 kGy gamma radiation and allowed to recover in TGY medium as described earlier (44). Different postirradiation recovery (PIR) fractions and controls were collected, washed with 70% ethanol, and snap-frozen in liquid nitrogen before storage at −70°C overnight. The levels of FtsA (monitored as FtsA-C18), FtsZ, and RqkA from the wild-type strain and of only FtsZ from the *rqkA* mutant during PIR were determined in irradiated and unirradiated cells of D. radiodurans by immunoblotting using monoclonal antibodies against T18, polyclonal antibodies against FtsZ, and polyclonal antibodies against RqkA, respectively, as described earlier (30). In brief, frozen cells were thawed on ice, suspended in lysis buffer {15 mM Tris-HCl (pH 7.6), 100 mM NaCl, 0.5 mM EDTA, 10% glycerol, 2% 3-[(3-cholamidopropyl)-dimethylammonio]-1-propanesulfonate (CHAPS), 1 mM PMSF, protease inhibitor cocktail}, and incubated further on ice for 1 h. Cells were sonicated, clear cell extract was obtained by centrifugation at 22,000 × *g* for 45 min at 4°C, and total protein content was estimated by Bradford assay. The supernatants of different fractions were precipitated by the use of acetone at −20°C overnight. Protein pellets containing similar amounts of protein were separated on a 10% SDS-PAGE gel and analyzed by Western blotting using antibodies as described above. Signals were detected using anti-mouse or anti-rabbit secondary antibodies conjugated with alkaline phosphatase and the color reaction substrates NBT and BCIP (Roch Biochemicals, Germany). Band intensities were quantified by the use of Image J2.0 software and plotted with standard deviations in GraphPad Prism5.

For *in vivo* phosphorylation of FtsZ and FtsA in D. radiodurans, the His-FtsA-expressing cells and His-FtsZ-expressing cells along with the vector control were subjected to 6 kGy gamma radiation and allowed to recover for 3 h in TGY medium as described earlier (44). At 3 h, cells were collected along with the respective SHAM controls and processed for preparation of cell extract and immunoprecipitation using polyhistidine antibodies as described above. Immunoprecipitates were separated on a 10% SDS-PAGE gel, blotted onto a PVDF membrane, and probed with polyclonal phospho-Ser/Thr antibodies (Cell Signaling Technology, Inc., USA) as described earlier (28). Similarly, the level of P-FtsZ during PIR was checked in the wild-type strain as well as the *rqkA* mutant. In brief, D. radiodurans cells expressing native FtsZ were irradiated by the use of 6 kGy gamma radiation and allowed to recover in TGY medium as described elsewhere. Different PIR fractions and the corresponding SHAM controls were collected and processed for coimmunoprecipitation using anti-FtsZ antibodies as described above. Immunoprecipitates were separated on a 10% SDS-PAGE gel, blotted onto a PVDF membrane, and probed with polyclonal phospho-Ser/Thr antibodies (Cell Signaling Technology, Inc., USA) as described earlier (28). Signals were detected using anti-rabbit secondary antibodies conjugated with alkaline phosphatase and the color reaction substrates NBT and BCIP (Roch Biochemicals, Germany). Band intensities were quantified by the use of ImageJ 2.0 software and plotted using GraphPad Prism5.

### GTPase activity measurement.

GTPase activity was measured using the malachite green assay and thin-layer chromatography (TLC) as described earlier (30, 31). For the malachite green assay, purified FtsZ or P-FtsZ (5 µM) was preincubated in PB buffer [25 mM piperazine-*N*,*N*′-bis(2-ethanesulfonic acid) (PIPES)-HCl (pH 7.5), 50 mM KCl] with 2 mM Mg^2+^ at 37°C for 10 min. To this, 5 µM FtsA was added in different combinations and further incubated for 10 min at 37°C. For measuring GTPase activity, the mixture was incubated for 10 min with 1 mM GTP. Reactions were stopped with 200 µl of freshly prepared malachite green reagent, and then the final volume was increased to 1 ml with the addition of distilled water. The release of inorganic phosphate was measured as described earlier (51). Absorbance at 630 nm was measured against the buffer control and normalized with a protein control that contained all components except GTP as well as with a GTP control that contained all components except protein. The amount of P_i_ released per minute per unit amount of enzyme was calculated using linear standard curve fitting and GraphPad Prism software. [α-^32^P]GTP was used for TLC, and the release of [α-^32^P]GDP was measured as described earlier (31). In brief, purified recombinant FtsZ or P-FtsZ (5 µM) was incubated in the absence and presence of 5 µM FtsA in PB buffer with 2 mM Mg^2+^ at 37°C for 10 min. To this, [^32^P]αGTP substrates (30 nM) were added. Reaction mixtures were further incubated at 37°C for 10 min. The reaction was stopped by addition of 10 mM EDTA. Further, 1 µl of the reaction mixture was spotted on polyethyleneimine (PEI)-cellulose F^+^ TLC sheets. Spots were air-dried, components were separated on a solid support in a buffer system in 0.75 M KH_2_PO_4_/H_3_PO_4_ (pH 3.5), and an autoradiogram was developed. Band intensities of samples were determined densitometrically, and the ratios of GDP to GTP were calculated and plotted.
