# Supplementary material for: Phosphorylation of FtsZ and FtsA by a DNA Damage-Responsive Ser/Thr Protein Kinase Affects Their Functional Interactions in Deinococcus radiodurans
Source: mSphere. 2018 Jul 18;3(4):e00325-18. doi: 10.1128/mSphere.00325-18 (PMC6052341; doi:10.1128/mSphere.00325-18)
Supplement: FIG S1 [file sph004182589sf1.pdf]

Harvard Medical School

Taplin Biological Mass Spectrometry Facility

Sample : FtsZ-P (tracking number 38946) database search results : data link

MAEEAAM**S#**AIHSPLLER and GGYDQGGYG**S#**GLVR- sites confidently assigned

1 MQAARIRVIGLGGAGNNAVNRMIESGLEGVEFIAGNTDAQVLAKSHAEVRIQLGDRLTRG 60

61 LGAGADPKVGEEAAVEDRDRIKEYLDDTDMLFITAGMGGGTGTGSAPVVAEIAREMGILT 12[illegible]

121 VAIVTRPFKFEGPKRMRVAEEGMSKLADRVDGMIVVNNEKLLTAVDKKVSFREAFLIADR 18[illegible]

235

181 VLYYGVKGISDVINVEGMINLDFADVRNLLANSGTVLMGIGAGRGDKMAEEAAM**S**AIHSP 24

241 LLERGIEGARRILVNVTGGYDLSMTDANEIVEKIREATGFDDPDILFGITPDEAAGDEVR 30

335

301 VTVIATGFGDNTYAAPLGGVVSSGRGGYDQGGYG**S**GLVRPVRGGQGGLGGGSSYDPKD3

Figure S1
