# Supplementary figures and images for: Phosphorylation of FtsZ and FtsA by a DNA Damage-Responsive Ser/Thr Protein Kinase Affects Their Functional Interactions in Deinococcus radiodurans
Source: mSphere. 2018 Jul 18;3(4):e00325-18. doi: 10.1128/mSphere.00325-18 (PMC6052341; doi:10.1128/mSphere.00325-18)

(A)


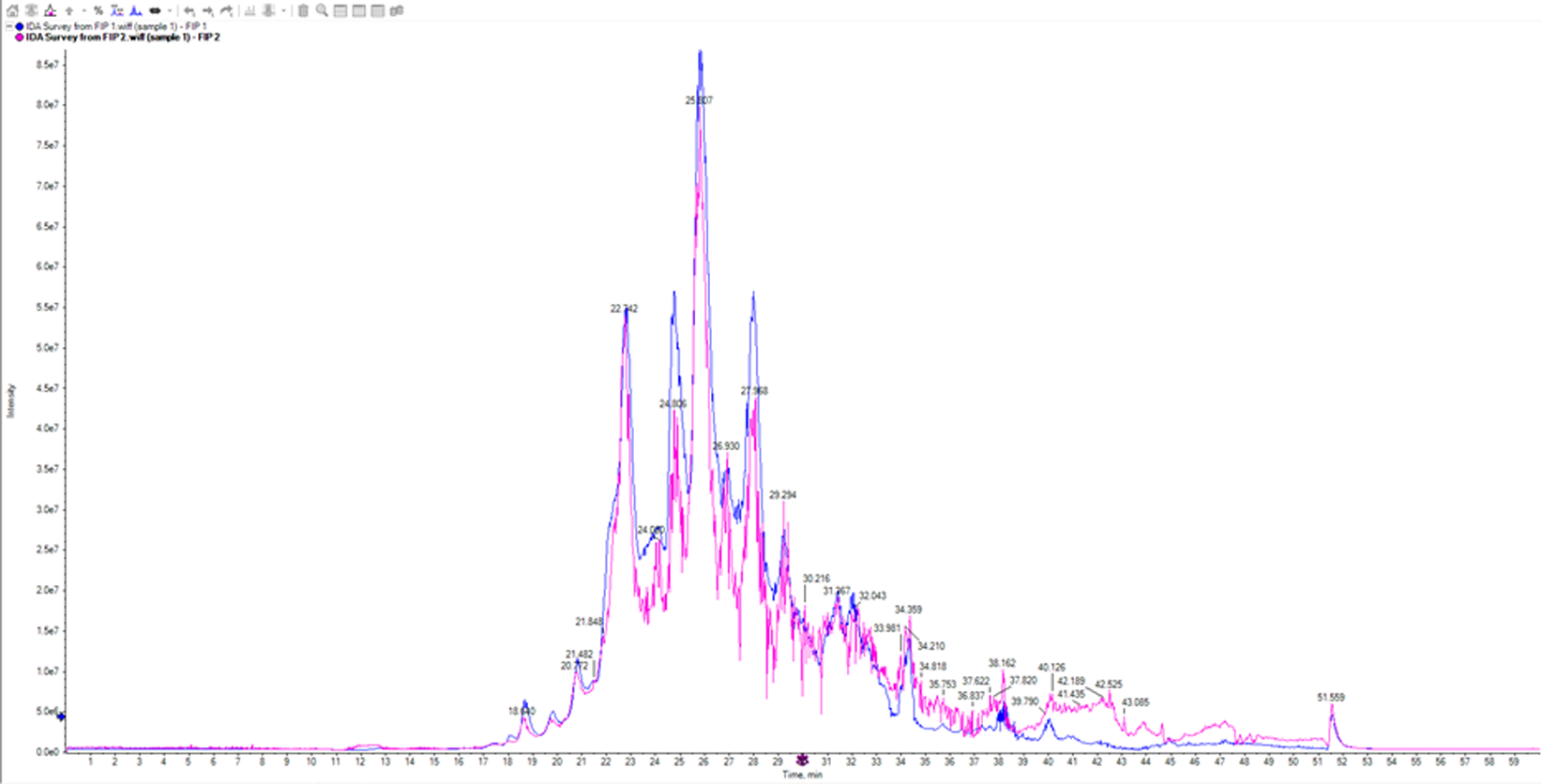


(B)

**Figure S2**

Supplement: FIG S2 [file sph004182589sf2.doc]

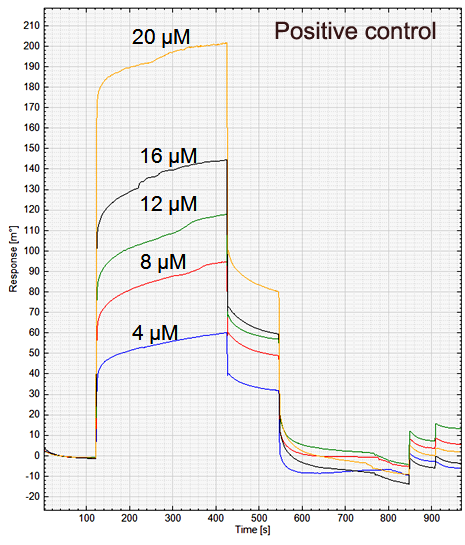


**Figure S7**

Supplement: FIG S7 [file sph004182589sf7.docx]

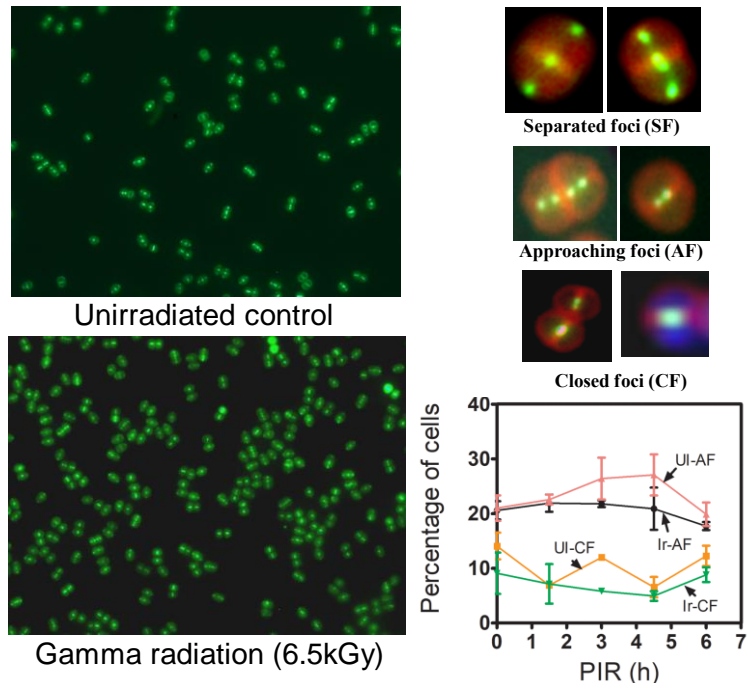

Fig S9

Supplement: FIG S9 [file sph004182589sf9.pdf]
