# Supplementary material for: Phosphorylation of FtsZ and FtsA by a DNA Damage-Responsive Ser/Thr Protein Kinase Affects Their Functional Interactions in Deinococcus radiodurans
Source: mSphere. 2018 Jul 18;3(4):e00325-18. doi: 10.1128/mSphere.00325-18 (PMC6052341; doi:10.1128/mSphere.00325-18)
Supplement: FIG S8 [file sph004182589sf8.docx]

**Figure S8**
