# Supplementary material for: Phosphorylation of FtsZ and FtsA by a DNA Damage-Responsive Ser/Thr Protein Kinase Affects Their Functional Interactions in Deinococcus radiodurans
Source: mSphere. 2018 Jul 18;3(4):e00325-18. doi: 10.1128/mSphere.00325-18 (PMC6052341; doi:10.1128/mSphere.00325-18)
Supplement: TABLE S1 [file sph004182589st1.doc]

**Table S1. Primers, bacterial strains, and plasmids used in this study**

| **Primer Name** | **Oligonucleotide Sequences** | | **Purpose/Plasmid** | |
| --- | --- | --- | --- | --- |
| FtsANdF | 5’ GGAATTC CATATG ATGAGAGAAAACAGCATC 3’ | | pFTSA, p21FTSA | |
| FtsABmR | 5’ GCGGATCC TCAGAACCAGTCGCGGAAC 3’ | |
| 2518F | 5’GGAATTCCATATG CCGCTGACCCCTGGAACCCT3’ | | pVHRqkA | |
| 2518R | 5’GGCCTCGAGCTACCCTTCCTGCTCGCT3’ | |
| BTHF(PV) | 5’ GGAATTCCATATGACCATGATTACG 3’ | | pVHSFtsA18 | |
| BTHR(PV) | 5’ GGCCTCGAGCATATTACTTAGTTA 3’ | |
| pETHisFw | 5’ AAAAGTACTGGGCCCATGGGCAGCAGCCAT 3’ | | pRadHisFZ, pRadHisFA | |
| pETHisRw | 5’ GCCTTAAGTCTAGATATCTCAGTGGTGGTG 3’ | |
| **Bacterial strains** | | **Genotype** | **Source** | |
| *D. radiodurans* R1 | | Wild type strain ATCC13939 | Lab stock | |
| *E. coli* MG1655 | | Wild type | Lab stock | |
| *E. coli* Novablue | | *end*A1 *hsd*R17*(r _K12_ − m _K12_ ^+^) sup*E44 *thi-1 rec*A1 *gyr*A96 *rel*A1 *lac*F’'*[pro*A^+^B*^+^ lac*I^q^ *Z∆*M15*::*T*n*10 ] (Tet^R^) | NEB Inc., | |
| *E. coli* BTH 101 | | F^-^, *cya-*99*, ara*D139*, gal*E15*, gal*K16*, rps*L1 (Str ^r^)*, hsd*R2*, mcr*A1*, mcr*B1 | 1 | |
| *E. coli* BL21(DE3) | | *fhu*A2 *(lon)omp*T *gal*(λDE3)*(dcm) ∆hs*dS | Lab stock | |
| *E. coli* BTH559 | | *E. coli* BTH 101 strain harboring shuttle vector pVHS559 (Str^R^ and Spec^R^) | This study | |
| *E. coli* BTHRqkA | | *E. coli* BTH 101 strain harboring shuttle vector pVHSRqkA (Str^R^ and Spec^R^) | This study | |
| **Plasmids** | | | | |
| **Names** | | **Characteristics** | **Source** | **MW of**  **protein** |
| pUT18 | | pUC19 derivative, MCS at N-terminal of T18 fragments of adenylate cyclase, ~3 kb, AmpR | 1 | 18 kDa |
| pKNT25 | | pSU40 derivative, MCS at N-terminal of T25 fragment of adenylate cyclase, ~3.4 kb, KanR | 1 | 25 kDa |
| pUTDFA | | pUT18 carrying dr*ftsA* at *Bam*HI and *Kpn*I | 2 | 72 kDa |
| pKNDFZ | | pKNT25 carrying dr*ftsZ* at *Bam*HI and *Kpn*I | 2 | 64 kDa |
| pUTEFA | | pUT18 carrying *E.coli* *ftsA* at *Xba*I and *Bam*HI | 2 | 63 kDa |
| pKNTEFZ | | pKNT25 carrying *E.coli* *ftsZ* at *Xba*I and *Bam*HI | 2 | 65 kDa |
| pVHS559 | | A shuttle vector between *D. radiodurans* and *E. coli* (Spec^R^) | 3 | - |
| pVHSRqkA | | pVHS559 carrying *rqkA* at *Nde*I and *Xho*I (Spec^R^) | This study | 71 kDa |
| pFTSA | | pET28a (+) carrying dr*ftsA* at *Nde*I and *Bam*HI | This study | 54 kDa |
| p21FTSA | | pET21a (+) carrying dr*ftsA* at *Nde*I and *Bam*HI | This study | 54 kDa |
| pFTSZdr | | pET28a (+) carrying dr*ftsZ* at *Nde*I and *Bam*HI | 4 | 40 kDa |
| pET2518 | | pET28a(+) carrying *rqkA* at *Nde*I and *Bam*HI | 5 | 76 kDa |
| pVHSFtsA18 | | pVHS559 carrying FtsA-C18 from pUTDFA at *Nde*I & *Xho*I | This study | 72 kDa |
| pRADgro | | pRAD1 carrying 261bp *Bgl*II-*Xba*I fragment of promoter (Pgro) from *D. radiodurans* | 6 | - |
| pRadHisFA | | pR**A**Dgro carrying coding sequence of (his)6- *ftsA* from pFTSA at *Apa*I and *Xba*I | This study | 54 kDa |
| pRadHisFZ | | pRADgro carrying coding sequence of (his)6- *ftsZ* from pFTSZdr at *Apa*I and *Xba*I | This study | 40 kDa |
| pRAD2518 | | pRADgro carrying *rqkA* at *Apa*I and *Xba*I | 7 | 71 kDa |

1. Saalbach G, Hempel AM, Vigouroux M, Flärdh K, Buttner MJ, Naldrett, M. 2013. Determination of phosphorylation sites in the DivIVA cytoskeletal protein of *Streptomyces coelicolor* by targeted LC–MS/MS. J. Proteome Res. 12:4187–4192.

2. Modi KM, Misra HS. 2014. Dr-FtsA, an actin homologue in *Deinococcus radiodurans*

differentially affects Dr-FtsZ and Ec-FtsZ functions in vitro. PLoS ONE 9:e115918.

3. Kreuzer KN. 2013. DNA damage responses in prokaryotes: regulating gene expression, modulating growth patterns, and manipulating replication forks. Cold Spring Harb. Perspect. Biol. 5: a012674.

4. Modi KM, Tewari R, Misra HS. 2014. FtsZDr, a tubulin homologue in radioresistant bacterium *Deinococcus radiodurans* is characterized as a GTPase exhibiting polymerization/depolymerization dynamics in vitro and FtsZ ring formation in vivo. Int. J.Biochem. Cell. Biol. 50:38-46.

5. Rajpurohit YS, Misra HS. 2010. Characterization of a DNA damage-inducible membrane protein kinase from *Deinococcus radiodurans* and its role in bacterial radioresistance and DNA strand break repair. Mol. Microbiol. 77:1470–1482.

6. Kang CM, Abbott DW, Park ST, Dascher CC, Cantley LC, Husson RN. 2005. The *Mycobacterium tuberculosis* serine/threonine kinases PknA and PknB: substrate identification and regulation of cell shape. Genes Dev.19:1692–1704.

7. Rajpurohit YS, Misra HS. 2013. Structure-function study of deinococcal serine/threonine protein kinase implicates its kinase activity and DNA repair protein phosphorylation roles in radioresistance of *Deinococcus radiodurans*. Int. J. Biochem. Cell. Biol. 45:2541-2552.
